# Operational and organizational variation in determinants of policy implementation success: the case of policies that earmark taxes for behavioral health services

**DOI:** 10.1186/s13012-024-01401-8

**Published:** 2024-10-31

**Authors:** Jonathan Purtle, Nicole A. Stadnick, Amanda I. Mauri, Sarah C. Walker, Eric J. Bruns, Gregory A. Aarons

**Affiliations:** 1 Department of Public Health Policy and Management, New York University School of Global Public Health, New York, NY USA; 2 Department of Psychiatry, University of California, San Diego, La Jolla, CA USA; 3 Altman Clinical and Translational Research Institute Dissemination and Implementation Science Center, University of California San Diego, La Jolla, CA USA; 4 Child and Adolescent Services Research Center, San Diego, CA USA; 5 Department of Psychiatry and Behavioral Sciences, University of Washington, Seattle, WA USA

## Abstract

**Background:**

Research on determinants of health policy implementation is limited, and conceptualizations of evidence and implementation success are evolving in the field. This study aimed to identify determinants of perceived policy implementation success and assess whether these determinants vary according to: (1) how policy implementation success is operationally defined [i.e., broadly vs. narrowly related to evidence-based practice (EBP) reach] and (2) the role of a person’s organization in policy implementation. The study focuses on policies that earmark taxes for behavioral health services.

**Methods:**

Web-based surveys of professionals involved with earmarked tax policy implementation were conducted between 2022 and 2023 (*N* = 272). The primary dependent variable was a 9-item score that broadly assessed perceptions of the tax policy positively impacting multiple dimensions of outcomes. The secondary dependent variable was a single item that narrowly assessed perceptions of the tax policy increasing EBP reach. Independent variables were scores mapped to determinants in the Exploration, Preparation, Implementation, and Sustainment (EPIS) framework. Multiple linear regression estimated associations between measures of determinants and policy implementation success.

**Results:**

Perceptions of tax attributes (innovation determinant), tax EBP implementation climate (inner-context determinant), and inter-agency collaboration in tax policy implementation (outer-context and bridging factor determinant) were significantly associated with perceptions of policy implementation success. However, the magnitude of associations varied according to how success was operationalized and by respondent organization type. For example, the magnitude of the association between tax attributes and implementation success was 42% smaller among respondents at direct service organizations than non-direct service organizations when implementation success was operationalized broadly in terms of generating positive impacts (β = 0.37 vs. β = 0.64), and 61% smaller when success was operationalized narrowly in terms of EBP reach (β = 0.23 vs. β = 0.59). Conversely, when success was operationalized narrowly as EBP reach, the magnitude of the association between EBP implementation climate and implementation success was large and significant among respondents at direct service organizations while it was not significant among respondents from non-direct service organizations (β = 0.48 vs. β=-0.06).

**Conclusion:**

Determinants of perceived policy implementation success may vary according to how policy implementation success is defined and the role of a person’s organization in policy implementation. This has implications for implementation science and selecting policy implementation strategies.

**Supplementary Information:**

The online version contains supplementary material available at 10.1186/s13012-024-01401-8.

Contributions to the literature

- The current study contributes to limited knowledge focused on determinants of policy implementation and scholarship about broadening definitions of evidence and implementation success in the field of implementation science.
- Attributes of earmarked tax policies, tax policy EBP implementation climate, and frequency of inter-agency collaboration in tax policy implementation are significant determinants of tax policy implementation success and could be targeted by policy implementation strategies.
- Determinants of policy implementation success vary according to how policy implementation success is operationally defined (broadly vs. narrowly related to EBP reach) and the role of a person’s organization in policy implementation the process.
- Linking policy implementation determinants to the EPIS framework advances understanding of how implementation science frameworks can advance the study of policy implementation.

## Background

 There have been recent policy-focused conceptual and methodological advances in the field of implementation science in health [1–20], as well as empirical research on disseminating research evidence to policymakers and the use of research evidence in policymaking [21–31]. Yet empirical work on health policy implementation (e.g., roll out after a policy has been enacted) within this field remains limited. More specifically, little quantitative work has focused on the *determinants* of policy implementation success. Identifying determinants of implementation success is important because it provides an empirical basis for the selection and tailoring of implementation strategies [32, 33], and can also inform decisions about policy development and initial implementation processes. However, in order to identify determinants of policy implementation success, “success” must first be operationally defined. This can be challenging in policy-focused implementation science.

Operationally defining what constitutes successful implementation of a policy is arguably less straightforward than defining what constitutes successful implementation of a clinical or programmatic intervention [34–36]. This is because clinical/programmatic interventions are deliberately designed with narrow goals of affecting a small number of outcomes at patient- and provider-levels. Before becoming the focus of an implementation science endeavor, interventions have usually demonstrated effectiveness at improving patient outcomes and are classified as an “evidence-based practice” (EBP). As such, measures directly related to an EBP’s delivery (e.g., adoption, reach, fidelity) are clear-cut indicators of implementation success [37–39]. 

In contrast, policies usually have broad goals and seek to affect a wide range of politically desirable outcomes—many of which are loosely specified [40]. Furthermore, the concept of “evidence-based” does not transfer neatly from interventions to policies because it is rarely feasible or ethical to randomize people to policy exposures [34–36]. The concept of “evidence-informed” better aligns with policy, but offers little utility in establishing indicators of policy implementation success. Policies are occasionally designed with the explicit goal of increasing the reach of EBPs [41, 42]. However, while defining policy implementation success in terms of EBP reach is well-aligned with how implementation success has traditionally been operationalized in implementation science, such EBP-focused policies have been critiqued as short-sighted and misaligned with the realities of policy implementers and consumers of services [43]. 

Within the context of increasing interest in health policy implementation science [1–20], complexities related to operationalizing policy implementation success, and calls for the field of implementation science to refine concepts of evidence [44, 45], the current study assesses if and how determinants of policy implementation success vary according to how implementation success is operationally defined and the role of a person’s organization in the policy implementation process. The study focuses on the implementation of policies that earmark tax revenue for mental health and substance use (i.e., behavioral health) services in the United States. We provide a brief overview of these policies before introducing our research aims and methodological approach.

## Policies that Earmark Tax Revenue for Behavioral Health Services

An earmarked tax is one placed on a specific base (e.g., goods, property, income) for which revenue is dedicated to a specific purpose [46–48]. Detailed descriptions of earmarked tax policies for behavioral health services and the larger policy implementation study protocol are provided elsewhere [49–55]. As reported in a 2019 commentary, two U.S. states—California and Washington—adopted high-profile policies which earmarked tax revenue for behavioral health services in 2005 [50]. A subsequent legal mapping study published in 2023 identified 207 policies in the United States that earmarked tax revenue for behavioral health services and found that the number of jurisdictions adopting these policies has steadily increased [52]. The study also found that the taxes generate about $3.57 billion annually and that approximately 30% of the U.S. population lives in a jurisdiction with such a tax.

These tax policies—95% of which are at city or county levels of government—vary significantly across jurisdictions in terms of their goals, designs, and the types of organizations involved with policy implementation [52]. The goals of these policies are typically broad and relate to outcomes such as increasing funding for and access to behavioral health and social services (regardless of whether these services are designated as EBPs), decreasing stigma about behavioral issues, and improving behavioral health outcomes at the population-level.

There is some evidence that these earmarked tax policies may positively affect implementation and effectiveness outcomes. For example, evidence from evaluations of California’s earmarked tax policy suggest that it may reduce death rates [14, 15] and improve the reach and sustainment of behavioral health services [56–58]. Descriptive survey-based and qualitative evidence from professionals involved with earmarked tax policy implementation across a range of jurisdictions suggests that the policies produce benefits such as increasing funding for behavioral health services, supporting flexibility in spending to meet local service needs, and potentially increasing the reach of EBPs [53, 54]. However, no prior research has focused on identifying determinants of successful implementation of these policies.

### Study aims

The current study seeks to generate an evidence base related to implementation determinants of policies that earmark tax revenue for behavioral health services, and to advance research on determinants of policy implementation outcomes more broadly. Specifically, the study seeks to achieve the following aims as they relate to policies that earmark tax revenue for behavioral health services:

1. Identify determinants of perceived policy implementation success,
2. Assess how these determinants vary when policy implementation success is operationally defined broadly and multi-dimensionally versus narrowly related to EBP reach, and.
3. Explore how these determinants vary between professionals at organizations that do versus do not provide direct services with earmarked tax revenue.

To achieve these aims, we analyzed survey data from 272 public agency and community organization professionals involved with earmarked tax policy implementation across seven U.S. states.

## Methods

The methods for the larger policy implementation study and pre-specified analyses are detailed in the study protocol [49]. 

## Conceptual Framework

**Fig. 1:**
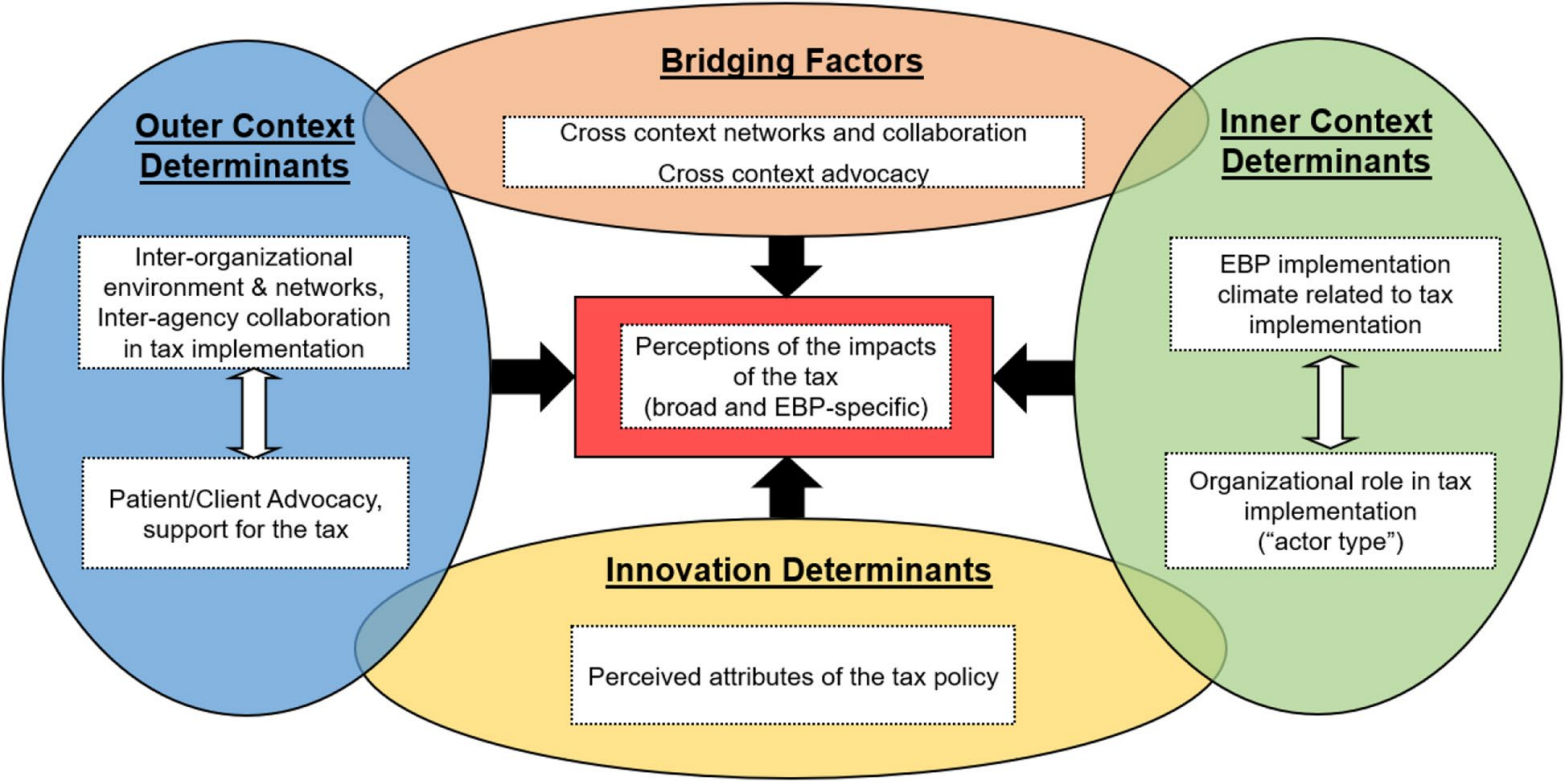
Adapted EPIS Conceptual Framework

The current study was informed by the Exploration, Preparation, and Sustainment (EPIS) framework [59], modified with recommendations for using the framework in policy-focused work by Crable and colleagues (Fig. 1) [14]. EPIS informed the selection of constructs assessed in the survey and guided data analysis related to determinants of earmarked tax policy implementation success. More details about these constructs and their measurement are provided below.

## Data

Study data originated from web-based surveys of government agency and community organization professionals involved with the implementation of policies that earmark taxes for behavioral health services. These include professionals involved with decision making, monitoring, and evaluating the taxes as well as professionals who provide and/or oversee direct services funded with tax revenue. Survey respondents were in positions such as, but not limited to, tax coordinators, leaders of state and county behavioral health agencies, service organizations that receive tax revenue, and members of tax advisory boards.

All jurisdictions in the United States that had policies earmarking taxes for behavioral health as of 2022 were identified in the aforementioned legal mapping study [52]. The survey sample frame was created by identifying professionals potentially involved with earmarked tax policy implementation in seven states: California, Colorado, Illinois, Kansas, Missouri, Ohio, and Washington. The method used to create the sample frame is aligned with recommendations for identifying key constituents when conducting policy-focused implementation science [19]. Contact databases maintained by practice partners (e.g., state and county behavioral health professional associations) were obtained, public meeting minutes and agency/organization websites were reviewed, and databases of behavioral health officials (compiled by the research team for prior studies) were reviewed to identify the names, titles, e-mail addresses, and phone numbers of professionals potentially involved with earmarked tax policy implementation in the seven states [60–62]. 

Web-based surveys were e-mailed to 691 professionals with valid e-mail addresses. The surveys were distributed between September 2022 and June 2023. Up to eight personalized e-mails were sent with a unique survey link, and telephone follow-up was conducted. To capture the perspectives of professionals involved with earmarked tax policy implementation who were not included in the original sample frame, we also created an open (i.e., not unique) survey link that was circulated by our aforementioned practice partners. A $20 gift card for survey completion was offered.

The exact wording of the survey items are included as Supplemental File 1. The survey was piloted with subject matter experts and revised prior to fielding. Survey items were selected, adapted, and as needed developed based on key informant interviews, research about earmarked taxes [46–48, 50, 52, 63–72], policy-focused implementation science measures [15–17], and constructs in the EPIS framework [14]. Items were forced response, but because jurisdictions vary widely in their designs of earmarked tax policies and the types of organizations involved with policy implementation, every item had a “not applicable” response opinion.

The full analytic sample consisted of 272 respondents. The unique survey link was completed by 222 respondents, for a response rate of 32.1% which is consistent with or higher than recent state-wide surveys of behavioral health officials [60–62]. The median state-specific response rate across the seven states was 45%. The open survey link was completed by an additional 50 respondents.

## Dependent variables

The primary dependent variable was a *broad*,* multi-dimensional operationalization of perceived tax policy implementation success*. The measure was a composite score derived from nine items (see Supplemental File 1) that quantified the extent to which the tax was perceived as generating positive impacts in the respondent’s jurisdiction. The impacts assessed by these items spanned sociopolitical, systems-level, implementation, and population health outcomes. The items were developed based on key informant interviews conducted during an earlier phase of the study and informed by literature on the potential benefits and drawbacks of earmarked taxes [46–48, 50, 63–72]. The list order of the nine items was randomized across respondents to reduce the risk of order effect bias [73]. Respondents rated the extent to which they agreed with each statement about the earmarked tax’s impact on a 7-point Likert scale (1= “strongly disagree”, 7= “strongly agree”). Items describing negative impacts were reverse coded. Reponses were summed to create a variable which quantified the extent to which the tax was perceived as having positive impacts, with a higher score representing more positive perceived impacts (possible scoring range: 9–63, Cronbach’s alpha = 0.70).

The secondary dependent variable was a *narrow*,* EBP-specific operationalization of perceived tax policy implementation success*. The measure was a single item that assessed agreement with the statement that “The tax increases the number of people served by evidence-based practices” (i.e., increases EBP reach) (1= “strongly disagree”, 7= “strongly agree”). Single-item measures can be appropriate for constructs such as these that are narrow in scope [74]. 

### Independent variables

Five determinant constructs spanning three domains of the EPIS framework served as the independent variables.

## Inner context determinants of tax policy implementation

Two variables in this domain characterized organizational factors that might affect policy implementation outcomes. First, *implementation climate* related to the tax being used to support EBPs was assessed using a seven-item adapted version of the Educational Support for Evidence-based Practice sub-scale of the Implementation Climate Scale [75]. The wordings of the 5-point Likert scale items was adapted to be focused on “perceptions of how your organization uses evidence when making decisions about the implementation of the earmarked tax for behavioral health in your jurisdiction.” These items were summed to create a composite implementation climate score, with higher scores indicating a stronger implementation climate related to tax funding supporting EBPs (possible scoring range: 7–35, Cronbach’s alpha = 0.88).

Second, respondents’ perceptions of *their organization’s roles in tax policy implementation* was examined. This variable was assessed by asking respondents to identify all of the “actor types,” derived from Leeman et al.’s typology [76], that they felt accurately characterized their organization’s role in tax implementation (i.e., delivery system actors, support system actors, synthesis and translation system actors). Definitions of these actor types were provided in the survey, adapted to be focused on earmarked tax policy implementation [55]. Given that a delineating role in earmarked tax policy implementation is whether the organization provides direct services funded by tax revenue, we created a dichotomous variable that captured whether or not each respondent identified their organization as a delivery system actor. This was used as a stratifying variable in secondary analysis (detailed below).

## Outer context and bridging determinants of tax policy implementation

Two variables in this domain characterized perceptions of factors external to the respondent’s organization that could affect tax policy implementation. The selection of these variables was informed by a review of outer-context measures in behavioral health implementation science [17]. First, *inter-organizational environment & networks* was measured by assessing the frequency of inter-organizational collaboration between the respondent and six types of external organizations on issues related to implementation of the tax policy. “Collaboration,” a potential EPIS bridging factor, was defined in the survey (Supplemental file1). The types of organizations were: substance use organizations, mental health organizations, public health departments/primary care service organizations, education departments/schools, child welfare agencies/child protective services, and justice departments/police. These items were summed to create an aggregate measure of inter-agency collaboration in earmarked tax implementation, with higher scores indicating more collaboration (possible scoring range: 6–30, Cronbach’s alpha = 0.84). The same definition and measure was used in prior research and significantly associated with the frequency of using research evidence in behavioral health policy implementation [77]. 

Second, *patient/client advocacy*, also a potential EPIS bridging factor, was measured by five items that assessed the extent to which respondents perceived five external constituent groups (i.e., the general public, consumers of behavioral health services, state behavioral health agency officials, state elected officials, local elected officials) as “strongly supporting” the earmarked tax policy. These items were summed to create an aggregate measure of external support, with higher scores indicating greater support (possible scoring range: 5–35; Cronbach’s alpha = 0.78). This measure was conceptualized as an indicator of the EPIS outer sociopolitical context in which policy implementation occurs, which can influence downstream policy implementation [78, 32]. 

### Policy Innovation determinants

Drawing from the concept of Attributes of Innovations in Rogers’ Theory of the Diffusion of Innovations [79], ten items assessed perceptions of earmarked tax policy attributes in the respondent’s jurisdictions (EPIS innovation factors). These items spanned the five innovation dimensions proposed by Rogers—complexity, observability, trialability, compatibility, and relative advantage—and each was assessed by two items. These dimensions have been assessed in prior behavioral health policy research [80]. Respondents indicated their level of agreement with each statement. Items focused on negative attributes were reverse coded, and the items were summed to create an aggregate measure of extent to which the tax policy was perceived as having positive attributes. Higher scores indicated more positive perceptions of tax design (possible scoring range: 10–70, Cronbach’s alpha = 0.79).

### Analyses

Descriptive statistics were calculated for all variables. Independent sample, two-tailed t-tests compared mean ratings of policy implementation success and determinants variables, stratified by whether the respondent indicated that their organization did vs. did not provide direct services funded by tax revenue. Missing data (i.e., items for which respondents indicated “not applicable”) were excluded from analysis and a composite score was not calculated for a respondent if an item needed to calculate the score was missing. The number and percentage of respondents for whom a score was not calculated for each variable, stratified by organization type, is provided in Supplement File 2.

Separate multiple linear regression models estimated associations between independent variables (determinants) and the two dependent variables of perceived policy implementation successes. For both dependent variables, separate models were first run using data from all respondents together and then limited to respondents indicating that their organization did vs. did not provide direct services with tax revenue. This resulted in a total of six models. Interaction terms assessed if associations between policy implementation determinant and success variables were significantly moderated by whether the respondent’s organization provided direct services with tax revenue.

The variance inflation factor (VIF) was between 1.0 and 2.0 (mean = 1.42) for all independent variables in all six models, indicating the absence of multi-collinearity [81]. Assessment of the normality of the data revealed that the scores for all variables were negatively skewed at a threshold ≥ 0.40. Thus, the scores were log transformed when entered into the models. Models were mutually adjusted for all determinant variables as well as respondent state. Study results are reported in accordance with STROBE guidelines (see checklist).

## Results

Table 1 shows the professional and demographic characteristics of the sample. Over two-thirds (41.9%) of respondents had worked at their organization for ten or more years and the modal highest level of education was Master’s degree (54.8%). The sample predominantly identified as female (68.3%) and non-Hispanic white (82.3%).

**Table 1:** Characteristics of Survey respondents, Public Agency, and Community Organization Professionals Involved with Implementation of Taxes Earmarked for Behavioral Health Services (2022–2023)

Respondent Characteristics	*n*	%
State		
California	90	33.2
Washington	68	25.1
Ohio	56	20.7
Illinois	22	8.1
Colorado	20	7.4
Missouri	14	5.2
Kansas	1	0.4
Gender		
Female	168	68.3
Male	78	31.7
Non-binary	0	0
Race/ethnicity		
White, Non-Hispanic	205	82.3
Hispanic	23	9.2
Black or African American	16	6.4
Asian	12	4.8
Native American/Alaskan Native	3	1.2
Years worked at current organization		
< 1	8	2.9
1–2	27	9.9
3–5	57	21.0
6–9	43	15.8
≥ 10	114	41.9
Highest level of education		
High school	1	0.4
Some college	6	2.2
College degree	59	21.7
Master’s degree (e.g., MS, MA, MPH)	149	54.8
Doctoral degree (e.g., MD, PhD, JD)	34	12.5

As shown in Table 2, the mean rating of broad policy implementation success was 46.8 (highest possible score: 63, SD = 8.6) and the mean rating of narrow, EBP-specific policy implementation success was 5.7 (highest possible score: 7, SD = 1.5). There were no significant differences in these mean ratings between respondents at organizations that did versus did not provide direct services with tax revenue.

**Table 2:** Perceptions of policy implementation success and determinants among Public Agency and Community Organization Professionals Involved with Implementation of Taxes Earmarked for Behavioral Health Services (2022–2023)

	All			Respondent’s Organization Provides Direct Services with Tax Revenue (*n* = 116)			Respondent’s Organization Does Not Provide Direct Services with Tax Revenue (*n* = 107)			
	N	Mean	SD	n	Mean	SD	n	Mean	SD	p
Policy Implementation Success										
Broad, multi-dimensional policy implementation success	251	46.75	8.64	109	47.21	9.35	99	46.87	7.73	0.78
Narrow, EBP-specific policy implementation success	272	5.69	1.51	116	5.77	1.46	107	5.76	1.45	0.96
Inner Context Determinants										
Tax policy EBP implementation climate	197	26.05	6.15	101	26.56	5.97	70	25.53	6.25	0.28
Outer Context/Bridging Determinants										
Inter-organizational environment & networks, inter-agency collaboration in tax policy implementation	256	23.46	5.07	109	23.00	5.37	104	24.49	4.27	0.03
Patient/client advocacy, external support for tax policy	259	27.63	4.99	112	27.10	5.20	106	28.19	4.74	0.11
Innovation Determinant										
Positive attributes of tax policy design	241	49.59	9.72	108	47.72	9.93	96	51.82	9.17	0.00

EBP = evidence-based practice

Also shown in Table 2, the mean implementation climate score related to the tax being used to support EBPs was 26.1 (highest possible score: 35, SD = 6.2), and 52.0% of respondents reported being at organizations that provided direct services with earmarked tax revenue. Across outer-setting variables, the mean inter-agency collaboration score related to the tax policy was 23.5 (highest possible score: 30, SD = 5.1), and the mean peer-pressure score related to the tax policy was 27.6 (highest possible score: 35, SD = 5.0). In the innovation domain, the mean positive perceptions of tax policy attributes score was 49.6 (highest possible score: 70, SD = 9.7).

### Adjusted associations between determinants and broad policy implementation success

Table 3, Model 1 shows adjusted associations between determinant variables and perceptions of broad, multidimensional policy implementation success for the entire sample. Determinant variables in the model explained 54.2% (adjusted R^2^ = 0.542) of the variance of the broad, multidimensional policy implementation success score. Greater tax EBP implementation climate (β = 0.26, *p* < .001), greater inter-agency collaboration in tax implementation processes (β = 0.20, *p* = .02), and more positive perceptions of the tax policy’s attributes (β = 0.50, *p* < .001) were all positively and significantly associated with more favorable perceptions of broad policy implementation success. The interpretation of these coefficients is that, for example, after adjustment, a 1% increase in positive perceptions of the tax’s attribute score is associated with a 0.50% increase in positive perceptions of broad policy implementation success.

**Table 3:** Adjusted associations between perceptions of Broad, multi-dimensional policy implementation success and determinants among Public Agency and Community Organization Professionals Involved with Implementation of Taxes Earmarked for Behavioral Health Services (2022–2023)

	Model 1: All Respondents (*n* = 153)			Model 2: Respondent’s Organization Provides Direct Services with Tax Revenue (*n* = 87)			Model 3: Respondent’s Organization Does Not Provide Direct Services with Tax Revenue (*n* = 66)		
	β	SE	p	β	SE	p	β	SE	p
Tax policy EBP implementation climate	0.26	0.05	< 0.001	0.38	0.09	< 0.001	0.19	0.06	0.04
Org. provides direct services with tax revenue (yes/no)	0.16	0.01	0.02	-	-	-	-	-	-
Inter-organizational environment & networks, inter-agency collaboration in tax policy implementation	0.20	0.06	< 0.001	0.19	0.08	0.04	0.22	0.11	0.02
Patient/client advocacy, external support for tax policy	0.05	0.07	0.47	-0.01	0.10	0.95	0.06	0.11	0.53
Positive attributes of tax policy design	0.50	0.07	< 0.001	0.37	0.10	< 0.001	0.64	0.08	< 0.001
State	-0.04	0.00	0.60	-0.05	0.00	0.56	-0.01	0.00	0.88
Interactions									
EBP climate * Org. direct service role	0.96	0.10	0.19	-	-	-	-	-	-
Inter-agency collaboration * Org. direct service role	-0.39	0.13	0.69	-	-	-	-	-	-
Attributes of tax * Org. direct service role	-1.82	0.12	0.08	-	-	-	-	-	-
Model Fit Statistics									
Adjusted R^2^	0.542			0.522			0.643		
F	30.93			19.80			21.62		
F (Sig)	< 0.001			< 0.001			< 0.001		

EBP = evidence-based practice. Org = organization

All three of these determinants remained significantly associated with positive perceptions of broad policy implementation success in separate models when the sample was stratified by respondents from organizations that did versus did not provide direct services with tax revenue. However, the magnitude of these associations varied for EBP implementation climate and perceptions of the tax’s attributes, while it remained similar for inter-agency collaboration (Table 3, Models 2 and 3). The magnitude of the adjusted association between tax EBP implementation climate and perceptions of broad policy implementation success was 50% larger for respondents in organizations that provided direct services with tax revenue compared to those in organizations that did not (β = 0.38 vs. β = 0.19) (implementation climate * organization direct service role interaction term *p* = .19). Conversely, the magnitude of the adjusted association between positive perceptions of the tax’s attributes and policy implementation success was 42% smaller for respondents in organizations that provided direct services with tax revenue compared to those in organizations that did not (β = 0.37 vs. β = 0.64). (tax attribute * organization direct service role interaction term *p* = .08).

### Adjusted associations between determinants and narrow, EBP-Specific policy implementation success

Table 4, Model 1 shows adjusted associations between determinant variables and perceptions of narrow, EBP-specific policy implementation success for the entire sample. Determinant variables in the model explained 27.5% of the variance of the narrow, EBP-specific policy implementation success score (adjusted R^2^ = 0.275). Greater tax EBP implementation climate (β = 0.19, *p* = .02) and more positive perceptions of the tax policy’s attributes (β = 0.38, *p* < .001) remained significantly associated with perceptions of policy implementation success. However, inter-agency collaboration in tax implementation processes was no longer associated with perceptions of policy implementation success (β = 0.06, *p* = .48).

**Table 4:** Adjusted associations between perceptions of narrow, EBP-Specific policy implementation success and determinants among Public Agency and Community Organization Professionals Involved with Implementation of Taxes Earmarked for Behavioral Health Services (2022–2023)

	Model 1: All Respondents (*N* = 158)			Model 2: Respondent’s Organization Provides Direct Services with Tax Revenue (*n* = 91)			Model 3: Respondent’s Organization Does Not Provide Direct Services with Tax Revenue (*n* = 67)		
	β	SE	p	β	SE	p	β	SE	p
Tax policy EBP implementation climate	0.19	0.09	0.02	0.48	0.16	< 0.001	-0.06	0.09	0.57
Org. provides direct services with tax revenue (yes/no)	0.02	0.02	0.81	-	-	-	-	-	-
Inter-organizational environment & networks, inter-agency collaboration in tax policy implementation	0.06	0.11	0.48	0.01	0.14	0.94	0.09	0.16	0.40
Patient/client advocacy, external support for tax policy	0.11	0.13	0.18	-0.02	0.17	0.88	0.14	0.17	0.22
Positive attributes of tax policy design	0.38	0.11	< 0.001	0.23	0.17	0.04	0.59	0.13	< 0.001
State	-0.09	0.00	0.27	-0.14	0.01	0.13	-0.01	0.00	0.96
Interactions									
EBP climate * Org. direct service role	2.71	0.16	0.002	-	-	-	-	-	-
Inter-agency collaboration * Org. direct service role	0.30	0.23	0.80	-	-	-	-	-	-
Attributes of tax * Org. direct service role	-1.82	0.20	0.16	-	-	-	-	-	-
Model Fit Statistics									
Adjusted R^2^	0.275			0.320			0.404		
F	10.93			9.48			9.93		
F (Sig)	< 0.001			< 0.001			< 0.001		

EBP = evidence-based practice. Org = organization

When the sample was stratified by whether the respondent’s organization did or did not provide direct services with tax revenue (Table 4, Models 1 and 2), the magnitude of the adjusted association between tax EBP implementation climate and narrow, EBP-specific policy implementation success increased substantially among respondents from organizations that provided direct services with tax revenue (β = 0.48, *p* < .001) while the association was not significant among respondents from organizations that did not provide these services (β= -0.06, *p* = .57) (implementation climate * organization direct service role interaction term *p* = .002). Consistent with associations observed when policy implementation success was operationalized broadly, the magnitude of the adjusted association between positive perceptions of the tax policy’s attributes and perceptions of narrow, EBP-specific policy implementation success was 61% smaller among respondents in organizations that did provide direct services with tax revenue compared to those that did not (β = 0.23 vs. β = 0.59) (tax attribute * organization direct service role term *p* = .16).

## Discussion

Within the context of policies that earmark tax revenue for behavioral health services, this study sought to empirically evaluate the extent to which theoretically-informed determinants were associated with perceptions of policy implementation success and assess whether these determinants vary according to how policy implementation success is operationalized and the role that a person’s organization plays in the policy implementation process. When policy implementation success is operationalized broadly, we find that the EPIS innovation determinant of perceived attributes of the tax policy, the inner-context determinant of EBP tax policy implementation climate, and the outer-context and bridging factor determinant of inter-agency collaboration in policy implementation are consistently and significantly associated with perceptions of policy implementation success—although the magnitudes of these associations vary by whether or not the respondent’s organization provided direct services with tax policy revenue.

However, when policy implementation success is operationalized narrowly in terms of EBP reach, we find that determinants of success vary more dramatically between respondents from organizations that do as opposed to do not provide direct services with tax revenue. Specifically, for respondents employed by direct service organizations, positive perceptions of policy impact on EBP reach were primarily driven by EBP implementation climate. By contrast, for respondents working in non-direct service organizations, only perceived attributes of the tax policy were significantly associated with perceived implementation success. The finding that inter-agency collaboration was not significantly associated with perceptions of the earmarked tax policy increasing EBP reach, regardless of organization type, is somewhat inconsistent with prior work [77, 82–86] and warrants future research.

Variations in the determinants of policy implementation success have general implications for policy-focused implementation science, as well as specific implications for the implementation of policies that earmark tax revenue for behavioral health services. Regarding implications for policy-focused implementation science, observing that determinants of “implementation success” varies by how success is defined—and by different types of actors—underscores the complexity of defining and operationalizing implementation success in policy-focused work. Methods related to participatory policymaking and implementation planning processes—such as policy co-design [87, 88] and multi-criteria decision support tools [89–91]—could be used to help integrate the perspectives of diverse constituencies and formulate operational definitions of implementation success for specific policies. It is possible—if not probable—that the definitions of policy implementation success produced through these methods will go beyond the bounds of what have traditionally been considered implementation outcomes in the field of implementation science. Consistent with broadening conceptualizations of “evidence” within the field [44, 45], expanding definitions of policy implementation success beyond metrics anchored to EBPs could be important to centering equity and implementation practitioner perspectives in implementation science [18, 92–94]. These definitions of success can inform theory-driven selection of determinants that are targeted by policy implementation strategies that are selected in initial policy development and implementation processes and also deployed post-hoc after a policy has been rolled out.

Regarding implications specific to the implementation of policies that earmark tax revenue for behavioral health services, study findings suggest that strategies that target: (a) determinants related to attributes of tax policy design and/or (b) implementation climate are candidate strategies that may foster policy implementation success. Findings suggest that targeting the attributes of tax earmarked policy design may be especially important when policy implementation success is operationalized broadly and multidimensionally. Although features of policy design are typically conceptualized as fixed determinants in implementation science [12], the attributes of earmarked tax policies can be shaped when policy proposals are developed (e.g., King County, Washington sales tax earmarked for crisis services, April 2023 [95]) and modified through policy reforms (e.g., California Proposition 1, March 2024 [96]). Data from surveys and interviews about policies earmarking tax revenue for behavioral health services indicate that key policy attributes relate to flexibility in spending decisions to meet local needs, clarity about permissible uses of tax revenue, minimally burdensome administrative reporting requirements, and the ability to carry annual tax revenue across multiple years [53, 54]. 

The study suggests that EBP implementation climate may be the key determinant to target with implementation strategies at organizations that provide direct services with tax revenue when policy implementation success is operationalized narrowly in terms of EBP reach. Specific strategies—such as the leadership and organizational change for implementation (LOCI) intervention—have demonstrated effectiveness at improving EBP implementation leadership and climate and implementation outcomes, and clinical outcomes in community behavioral health service settings [96–100]. Funding for such implementation support could be codified in earmarked tax spending plans. Additional data from the study’s survey (presented elsewhere [55]) found that integration and implementation process strategies—both of which could affect implementation climate—were perceived as both acceptable and feasible strategies to use to increase the reach of EBPs with earmarked tax revenue.

### Limitations and considerations

The study’s findings should be considered within the context of its scope and limitations. First, it should be emphasized that the study sought to identify determinants of *perceptions* of policy implementation success as opposed to objective measures of policy implementation outcomes. It is possible that different determinants would have been identified if policy implementation outcomes were measured more objectively. However, it would be challenging, if not infeasible, to conduct a similar study with objective outcomes data related to these policies given limitations and inconsistencies in data availability at the local level in the United States. Assessing perceptions of policy implementation success is an approach to uniformly capture outcomes across a sufficiently large number of jurisdictions that vary in policy design and implementation context. It should also be emphasized that the sample was primarily comprised of administrative officials. Results could differ in a sample of elected policymakers (e.g., variables related to public support and political context may be stronger determinants).

Second, also related to heterogeneity in earmarked tax policies across jurisdictions, all survey items had a “not applicable” response opinion. This decision was made to enhance the quality of data and increase internal validity by not forcing respondents to select an answer to a question for which they have no opinion or experience to draw from. However, this approach increased “missingness” in the data (although the data are not technically missing because respondents indicated “not applicable”), which reduced the number of respondents with composite scores and size of the analytic samples. As such, some regression analyses may have been under-powered to identify statistically significant associations. Imputation for missing values would not be a logical approach because it would result in assigning ratings to items that respondents deliberately indicated were not applicable.

Third, although the survey response rate of 32.1% is consistent with recent state-wide surveys of behavioral health officials in the United States [60–62], the sample may not reflect the perspectives of all professionals involved with the implementation of policies that earmark tax revenue for behavioral health services. As described here and elsewhere [53, 54], the types of professionals involved with earmarked tax policy implementation vary across jurisdictions. Many individuals to whom the survey was sent replied and indicated that they did not complete the survey because they were not actively involved with tax policy implementation. Thus, the survey sample likely reflects those most engaged in local tax implementation and decision-making processes.

Fourth, although survey items were piloted and informed by theory (e.g., Rogers’s constructs of attributes of innovations [79], the EPIS framework [14]) and prior research about earmarked taxes [46–48, 52, 63–69], many items were newly developed or adapted for the survey. As identified in systematic reviews [15–17], there is a paucity of measures focused on policy implementation that have undergone robust psychometric testing.

## Conclusion

The determinants of perceptions of health policy implementation success vary according to how policy implementation success is operationally defined and the role of a person’s organization in policy implementation. Our study highlights the importance of thoughtfully specifying how policy implementation success is conceptualized and who has a say in defining intended impact. Such conceptual work can help identify implementation determinants to target with policy implementation strategies.

## Supplementary Information

Supplementary Material 1.

Supplementary Material 2.

Supplementary Material 3.

## Data Availability

The datasets used and/or analyzed during the current study are available from the corresponding author on reasonable request.
