# Supplementary material for: Operational and organizational variation in determinants of policy implementation success: the case of policies that earmark taxes for behavioral health services
Source: Implement Sci. 2024 Oct 31;19:73. doi: 10.1186/s13012-024-01401-8 (PMC11526668; doi:10.1186/s13012-024-01401-8)
Supplement: Supplementary file 2 — Supplementary Material 2. [file 13012_2024_1401_MOESM2_ESM.docx]

**Supplement 1. Survey Questions**

| 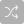 |
| --- |

Rate the extent to which you agree with the following statements about the **impacts** of the earmarked tax for behavioral health in your jurisdiction.

|  | **1= Strongly disagree** | **Strongly agree= 7** | Not Applicable |
| --- | --- | --- | --- |

|  | 1 | 2 | 3 | 4 | 5 | 6 | 7 |
| --- | --- | --- | --- | --- | --- | --- | --- |

| The tax increases funding for direct behavioral health/social services () | 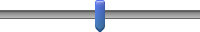 |
| --- | --- |
| The tax increases funding for improvements to behavioral health/social services systems () | 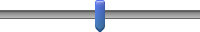 |
| The tax increases the number of people served by evidence-based practices () | 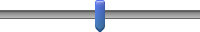 |
| The tax increases flexibility to address complex behavioral health/social serviced needs () | 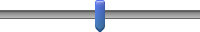 |
| The tax increases access to direct behavioral health/social services for people with the highest need () | 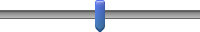 |
| The tax is increases public awareness about behavioral health issues () | 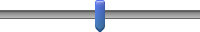 |
| The tax decreases stigma about behavioral health issues () | 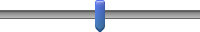 |
| The tax decreases funding from other sources (e.g., general county/state budgets) for behavioral health/social services () | 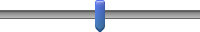 |
| The tax increases transparency about behavioral health/social services systems () | 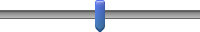 |

| 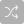 |
| --- |
|  |

Q26 Rate the extent to which agree with the following statements about the **characteristics** of the earmarked tax for behavioral health in your jurisdiction.

|  | **1= Strongly disagree** | **Strongly agree= 7** | Not Applicable |
| --- | --- | --- | --- |

|  | 1 | 2 | 3 | 4 | 5 | 6 | 7 |
| --- | --- | --- | --- | --- | --- | --- | --- |

| It is hard to understand what is and is not a permissible use of revenue from the tax () | 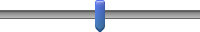 |
| --- | --- |
| It is complicated to satisfy reporting requirements related to using of revenue from the tax () | 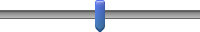 |
| The impact of the tax on the number of people who receive services is easy to observe () | 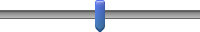 |
| The impact of the tax on the behavioral health status of communities is easy to observe () | 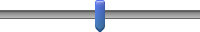 |
| The rules related to how revenue from the tax can be spent can be easily changed to address emergent needs () | 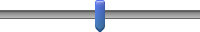 |
| The tax allows behavioral health service organizations to try new services assess whether they meet needs before taking the services to scale () | 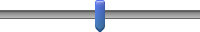 |
| The tax is flexible enough to allow behavioral health service organizations to meet the unique needs of the communities they serve () | 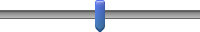 |
| The tax is compatible with the financing structures of behavioral health service organizations () | 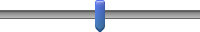 |
| It is better to have the tax than not () | 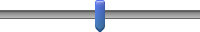 |
| The tax is better than alternative strategies to increase funding for behavioral health services () | 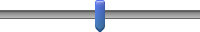 |

End of Block: Perceptions of the benefits and attributes of the tax

Start of Block: Outer context: Cosmopolitanism and Peer-pressure

Q10 *Next is a question about your perceptions of support for the earmarked tax for behavioral health in your jurisdiction among different groups.*

| 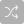 |
| --- |

Q11 Rate the extent to which you agree that there is **strong support for the tax** among….

|  | **1= Completely disagree** | **Completely agree= 7** | Not Applicable |
| --- | --- | --- | --- |

|  | 1 | 2 | 3 | 4 | 5 | 6 | 7 |
| --- | --- | --- | --- | --- | --- | --- | --- |

| The general public in my jurisdiction () | 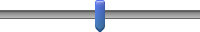 |
| --- | --- |
| Local elected officials in my jurisdiction () | 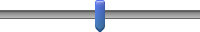 |
| Consumers of behavioral health services in my jurisdiction () | 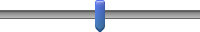 |
| State behavioral health agency officials in my state () | 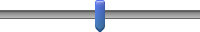 |
| State elected officials in my state () | 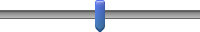 |

Q12 *Next is a question about how often you collaborate with external agencies on issues related to implementation of the earmarked tax.*
**By collaboration we mean the process by which government agencies come together and establish a formal commitment to working together to achieve common goals.**

| 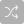 |
| --- |

Q13 Indicate how often you collaborate with each of the following on issues related to implementation of the earmarked tax.

|  | **1= Never** | **Very frequently= 5** | Not Applicable |
| --- | --- | --- | --- |

|  | 1 | 2 | 3 | 4 | 5 |
| --- | --- | --- | --- | --- | --- |

| Local substance use agency/direct service organizations () | 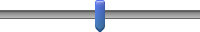 |
| --- | --- |
| Local mental health agency/direct service organizations () | 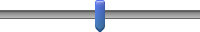 |
| Local public health department/primary care service organizations () | 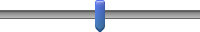 |
| Local education department/schools () | 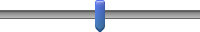 |
| Local child welfare agency/child protective services () | 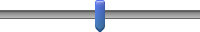 |
| Local justice department/police () | 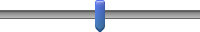 |

End of Block: Outer context: Cosmopolitanism and Peer-pressure

Start of Block: Inner-context, Imp Climate

Q20 *Next is a question about your perceptions of how your organization uses evidence when making decisions about the implementation of the earmarked tax for behavioral health in your jurisdiction.*

| 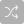 |
| --- |

Q21 Indicate the extent to which you agree with each of the statements below about your organization within the context of decisions about evidence-based practice and implementation of the earmarked tax for behavioral health in your jurisdiction.

|  | **1= Not at all** | **Very great extent= 5** | Not Applicable |
| --- | --- | --- | --- |

|  | 1 | 2 | 3 | 4 | 5 |
| --- | --- | --- | --- | --- | --- |

| One of this organization’s main goals is to use evidence-based practices effectively with earmarked tax revenue () | 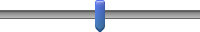 |
| --- | --- |
| People in this organization think that the implementation of evidence-based practices with earmarked tax revenue is important () | 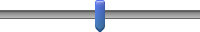 |
| Using evidence-based practices is a top priority in this organization when it comes to making decision about earmarked tax revenue () | 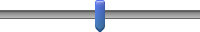 |
| This organization provides conferences, workshops, or seminars focusing on using earmarked tax revenue for evidence-based practices () | 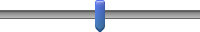 |
| This organization uses earmarked tax revenue to provide evidence-based practice trainings or in-services () | 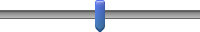 |
| This organization uses earmarked tax revenue to provide evidence-based practice training materials, journals () | 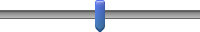 |
| Clinicians in this organization who use evidence-based practices are seen as clinical experts () | 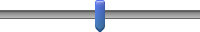 |

End of Block: Inner-context, Imp Climate

Start of Block: Strategies

*Final are a few demographic questions.*

| 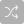 |
| --- |

Q3 Please indicate which categories most accurately describe **your organization’s** role within the context of implementing the earmarked tax for behavioral health in your jurisdiction. Select all that apply.

- Providing direct behavioral health and social services with tax revenue (1)
- Supporting system and capacity building efforts for organizations that provide direct behavioral health and social services with tax revenue (2)
- Reviewing evidence about promising approaches to using earmarked tax revenue and communicating this information to organizations that provide direct behavioral health and social services (3)

Q23 What is your gender?

- Female (1)
- Male (2)
- Non-binary (3)

Q24 What is your race/ethnicity (select all that apply)?

- Black or African American (1)
- White, Non-Hispanic (2)
- Hispanic (3)
- Native American/Alaskan Native (4)
- Asian (5)

Q25 In total, how many years have you worked at your organization?

- Less than one year (1)
- One to two years (2)
- Three to five years (3)
- Six to nine years (4)
- Ten or more years (5)

Q26 What is the highest level of education that you have completed?

- High school or GED (1)
- Some college (2)
- College degree (3)
- Master’s degree (e.g., MS, MA, MPH) (4)
- Doctoral degree (e.g., MD, PhD, JD) (5)

End of Block: Demographics

Start of Block: Incentive and snowball
