# Supplementary material for: Operational and organizational variation in determinants of policy implementation success: the case of policies that earmark taxes for behavioral health services
Source: Implement Sci. 2024 Oct 31;19:73. doi: 10.1186/s13012-024-01401-8 (PMC11526668; doi:10.1186/s13012-024-01401-8)
Supplement: Supplementary file 3 — Supplementary Material 3. [file 13012_2024_1401_MOESM3_ESM.docx]

**Supplemental Table: Missing Variable Data Across Key Constructs**

|  | All  (N=272) | | Respondent’s Organization Provides Direct Services with Tax Revenue  (n= 116) | | Respondent’s Organization Does Not Provide Direct Services with Tax Revenue  (n=107) | |
| --- | --- | --- | --- | --- | --- | --- |
| Measure | n missing | % missing | n missing | % missing | n missing | % missing |
| Policy Implementation Success | | |  |  |  |  |
| Broad, multi-dimensional policy implementation success | 21 | 7.7% | 7 | 6.0% | 8 | 7.5% |
| Narrow, EBP-specific policy implementation success | 0 | 0.0% | 0 | 0.0% | 0 | 0.0% |
| Inner Context Determinants | | |  |  |  |  |
| Tax policy EBP implementation climate | 75 | 27.6% | 15 | 12.9% | 37 | 34.6% |
| Outer Context/Bridging Determinants | | | |  |  |  |
| Inter-organizational environment & networks, inter-agency collaboration in tax policy implementation | 16 | 5.9% | 7 | 6.0% | 3 | 2.8% |
| Patient/client advocacy, external support for tax policy | 13 | 4.8% | 4 | 3.4% | 1 | 0.9% |
| Innovation Determinant | |  |  |  |  |  |
| Positive attributes of tax policy design | 31 | 11.4% | 8 | 6.9% | 11 | 10.3% |
